# Getting patients back for routine colorectal cancer screening: Randomized controlled trial of a shared decision‐making intervention

**DOI:** 10.1002/cam4.5172

**Published:** 2022-09-02

**Authors:** Karen R. Sepucha, Kathrene D. Valentine, Steven J. Atlas, Yuchiao Chang, Kathleen M. Fairfield, Jasmine Ha, Lauren Leavitt, Vivian Lee, Sanja Percac‐Lima, James M. Richter, Leigh Simmons

**Affiliations:** ^1^ Massachusetts General Hospital Boston Massachusetts USA; ^2^ Harvard Medical School Boston Massachusetts USA; ^3^ Maine Medical Center Portland Maine USA

**Keywords:** behavioral science, cancer education, colorectal cancer, screening

## Abstract

Thousands of colonoscopies were canceled during the initial surge of the COVID‐19 pandemic. As facilities resumed services, some patients were hesitant to reschedule. The purpose of this study was to determine whether a decision aid plus telephone coaching would increase colorectal cancer (CRC) screening and improve patient reports of shared decision making (SDM). A randomized controlled trial assigned adults aged 45–75 without prior history of CRC who had a colonoscopy canceled from March to May 2020 to intervention (*n* = 400) or usual care control (*n* = 400) arms. The intervention arm received three‐page decision aid and call from decision coach from September 2020 through November 2020. Screening rates were collected at 6 months. A subset (*n* = 250) in each arm was surveyed 8 weeks after randomization to assess SDM (scores range 0–4, higher scores indicating more SDM), decisional conflict, and screening preference. The sample was on average, 60 years old, 53% female, 74% White, non‐Hispanic, and 11% Spanish speaking. More intervention arm patients were screened within 6 months (35% intervention vs 23% control, *p* < 0.001). The intervention respondents reported higher SDM scores (mean difference 0.7 [0.4, 0.9], *p* < 0.001) and less decisional conflict than controls (−21% [−35%, −7%], *p* = 0.003). The majority in both arms preferred screening versus delaying (68% intervention vs. 65% control, *p* = 0.75). An SDM approach that offered alternatives and incorporated patients' preferences resulted in higher screening rates. Patients who are overdue for CRC screening may benefit from proactive outreach with SDM support.

## Graphical abstract

Many patients who had their colonoscopies cancelled or postponed due to the COVID‐19 pandemic were hesitant to reschedule. This randomized trial found that a brief shared decision making intervention significantly increased colorectal cancer screening uptake and improved the quality of screening decisions over usual care.

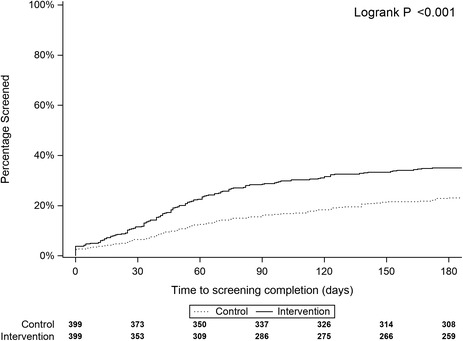

## 1 INTRODUCTION

Restrictions on elective procedures instituted in response to the initial surge of COVID‐19 cases in spring 2020 resulted in a sharp reduction in colonoscopies for colorectal cancer (CRC) screening both nationally and internationally.^1^, ^2^, ^3^, ^4^ After facilities resumed elective healthcare services, many faced additional challenges such as longer wait times due to backlogs, limits on volume due to new requirements for infection control, and patients' hesitance due to COVID‐19 worry.^5^, ^6^ Public opinion polls during 2020 showed that the majority of patients were unlikely to visit a hospital (62%) or specialist (64%), regardless of whether they lived in a COVID‐19 hotspot.^7^ If patients are unwilling to return to routine cancer screening, then these testing delays may have a significant impact on future CRC morbidity burden and mortality rates.

Informing patients about their CRC screening options, including home‐based stool tests and colonoscopy, and eliciting and addressing their concerns about testing may improve decisions and help increase overall screening rates. Patients report a strong desire for this type of an engaged, involved approach^8^; yet, studies suggest clinicians tend to recommend one screening test with little discussion of other options, falling short of the shared decision‐making (SDM) ideal.^9^, ^10^ Prior research has demonstrated the effectiveness of interventions such as patient decision aids and decision coaching to promote SDM.^11^, ^12^ Whether an SDM approach would work with patients who may be hesitant to seek care during the pandemic is not clear.

The purpose of this randomized controlled trial is to examine the effectiveness of an SDM approach for patients with a canceled or delayed screening colonoscopy during the COVID‐19 pandemic. The main hypotheses are that patients in the intervention arm will have higher uptake of screening, as well as higher SDM scores, less decisional conflict, and will be more likely to prefer screening (either colonoscopy or stool‐based test) compared with patients in the control arm.

## 2 METHODS

All study activities were approved by Mass General Brigham Institutional Review Board (activation date 6/2/2020) and the study is registered on ClinicalTrials.gov (#NCT04548531, release date 9/9/2020). Reporting follows the CONSORT guidelines and Standards for Universal Reporting of Decision Aid Evaluations (SUNDAE) guidelines.^13^, ^14^

### 2.1 Design

Randomized controlled trial where patient participants were randomized 1:1 into either intervention or control arms. The intervention mailings went out in four waves, about 2 weeks apart, between 10 September 2020 and 22 October 2020. Participants in the intervention arm received a decision worksheet in the mail followed by a call from a decision coach. A subset of participants across both arms was selected to receive a survey. Staff mailed a survey to the selected subset of control and intervention participants about 8 weeks after the intervention packet was mailed to their wave. The survey packet included a cover letter, an information sheet, an incentive ($10 gift card), the survey, and a return envelope. Patients were able to complete the survey by mail, online via REDCap, or over the phone with study staff. Staff made up to three reminder phone calls, sent a reminder mailing to nonresponders, and made up to three additional reminder calls to encourage survey completion. Of note, staff conducting the reminder calls were not the same as the decision coaches. Staff conducted chart review to collect screening tests completed within 6 months.

### 2.2 Setting and COVID impact

On 15 March 2020, all preventive and elective procedures, including colonoscopies, were prohibited as part of the executive orders issued by the Commonwealth of Massachusetts in response to the pandemic. Many clinical and nonclinical staff in the gastroenterology (GI) department were redeployed to other areas of the hospital to support COVID‐related care, while other staff took on new and additional roles within the practice to maintain clinical operations. Most colonoscopies were completely suspended during this time, referrals for screening colonoscopies were not processed, and patients who were overdue and would have been proactively contacted by GI to schedule a colonoscopy were not contacted. On 22 May 2020, the state relaxed the restrictions and elective procedures were again permitted, albeit with additional restrictions to reduce the risk of COVID‐19 transmission. Starting in June 2020, the main goal for the GI department was to contact patients and reschedule their colonoscopies, with a priority for high‐risk patients. Patients were contacted through the online patient portal, email, text message, or phone call to schedule their procedure. Patients were also able to call into the department to directly schedule a procedure.

### 2.3 Participants

On 2 June 2020, the coinvestigators from the gastroenterology department extracted a list of patients aged 45–75, with the preferred language of English or Spanish, who had a screening or surveillance colonoscopy that was canceled, who had a referral for a screening colonoscopy that had not been processed, or who should have been contacted by the GI department to schedule a screening colonoscopy but had not been due to COVID‐19 restrictions. Study staff removed patients who did not meet eligibility criteria, as outlined in Table 1. An additional review was conducted immediately prior to selecting the sample in mid‐September 2020 to exclude patients who had already completed or rescheduled their colonoscopy.

**TABLE 1:** Eligibility criteria for the patient sample

Eligible	Ineligible
Adults, age 45–75 Had screening or surveillance colonoscopy canceled due to COVID‐19 or was due for outreach from gastroenterology department to schedule routine screening or surveillance colonoscopy from 13 March to 30 May 2020.	Scheduled or due for diagnostic colonoscopy High risk for colorectal cancer as indicated by 1‐year follow‐up interval on prior test Prior history of colon or rectal cancer Unable to read or write in English or Spanish Scheduled or completed colonoscopy after 30 May 2020 and before selection into the study sample.

### 2.4 Interventions

A three‐page decision worksheet was mailed to participants, followed by a telephone session with a trained decision coach within 1–2 weeks. The decision worksheet presented the pros and cons of three options: (1) colonoscopy, (2) stool‐based test, and (3) delay screening until next year. Primary care physicians, gastroenterologists, patient partners, and decision scientists were involved in the development of the decision worksheet. We also had four patients, who had their colonoscopy canceled due to COVID, review the worksheet and provide feedback on acceptability, framing, and comprehensibility. The worksheet was edited by an expert in plain language, was translated into Spanish, and is available online.^15^

The coaching session was conducted by nonclinical research staff who were trained as coaches using Ottawa Decision Support coaching techniques.^16^ A decision coaching tutorial is available online.^17^ On the call, the coach reviewed the worksheet, answered questions, elicited the patient's preferences, and helped facilitate the scheduling of a preferred screening test or an appointment with a GI clinician (if needed based on the complexity of patients' situation and questions). Patients who requested a GI visit were referred to one of seven GI physicians or nurse practitioners who had attended a short, 30‐min training session led by study investigators to review SDM skills, with a focus on the pros and cons of stool‐based tests and logistics for ordering these tests. Coaches made one attempt to contact patients by phone to conduct the coaching session. One trained coach was a native Spanish speaker (and certified medical interpreter) and conducted the coaching sessions with Spanish‐speaking participants. Only one call attempt was made in an effort to keep the intervention more feasible.

### 2.5 Control

The control arm received the usual care. The GI department contacted patients due for a colonoscopy to schedule their procedure. Patients were also able to call the department directly to schedule.

### 2.6 Randomization and blinding

The study statistician used a computer random number generator to randomly select 800 eligible patients, assign each to intervention or control arm, and to one of four waves. All 800 patients were followed to track colon cancer screening tests completed and a subset in each wave was randomly selected to receive a survey to measure patient‐reported outcomes. The staff who entered the data from the paper surveys and who conducted chart review to collect screening were blinded to the assignment. The statistician analyzing the results was not blinded to the assignment.

### 2.7 Outcomes

Screening uptake: Study staff examined medical records to determine receipt of any CRC screening test for all subjects within 6 months.

The following measures were collected in the patient survey:

SDM Process Scale: Four‐item survey asks about the discussion of stool test, the pros and cons of colonoscopy, and patient's screening preference. Total scores range from 0 to 4 with higher scores indicating higher SDM.^18^ Patients who indicated that they did not talk with anyone about CRC screening received an SDM Process score of 0.

SURE scale: The brief four‐item version of the decisional conflict scale. A point is given for each “yes” response for total scores 0–4 and we report the percentage receiving the top score of 4, which indicates no decisional conflict.^19^

Screening preference: One item asked the patient's preferred approach to screening (with responses: colonoscopy, stool‐based test, delay screening, not sure).

Additional measures were collected to describe the sample and used as covariates including whether or not the patient had a CRC discussion with a healthcare provider in the past 2 months, PROMIS Scale v1.2‐Global Health Physical 2a,^20^ Single Item Literacy Screener,^21^ CRC screening and history, COVID‐19 worry, decision worksheet use, and decision coaching exposure. Basic demographics for the full sample were collected via chart review. Of note, the medical record collects one item that combines race and ethnicity; whereas the survey asked for ethnicity as a separate item from the race.

### 2.8 Sample size

The sample size of 800 was determined based on the screening uptake, with 800 participants, the study had 81% power to detect a difference of 10% in rates. For the survey, we assumed a 60% response rate and as a result, planned to invite about 500 patients (250 in each arm) to obtain 300 responses. With 300 survey responses, the study had 80% power to detect a difference of 0.32 standard deviation (SD) for the SDM Process score. Studies have found effect sizes ranging from 0.39 SD to 0.88 SD for SDM Process when comparing sites that used formal decision support (coaching or decision aids) and those that did not.^22^ For dichotomized survey outcomes (% SURE top score and % prefer screening), with 300 responses, we had 80% power to detect a 13% difference, from 70% to 83%.

### 2.9 Statistical methods

Sample demographics and characteristics were compiled and compared to evaluate the balance between the two arms. Responders and nonresponders to the survey were compared between arms to evaluate potential nonresponse bias.

The following hypotheses were evaluated using an intention‐to‐treat approach, and patients were analyzed based on their assigned arm.

1. Compared to the control group, patients in the intervention arm will be more likely to have a screening test within 6 months (the percentage of patients who had either stool test or colonoscopy in each arm compared using a Fisher Exact test). We also used the cumulative incidence function to compare time to screening completion.
2. Compared to the control group, patients in the intervention arm will report higher SDM scores (compared mean scores using a two‐sample *t*‐test).
3. Compared to the control group, patients in the intervention arm will (3a) be more likely to have a clear preference for colon cancer screening (either colonoscopy or stool‐based test) and (3b) have a less decisional conflict (i.e., a higher percentage of SURE top scores). We compared the percentage of patients with these outcomes between arms using chi‐square analyses.

In a prespecified analysis, we explored the heterogeneity of treatment effects to identify differential treatment effects among subgroups of patients stratified by sex as a biological variable, age, race/ethnicity, education, overall health, prior screening history, COVID worry, and interaction with decision coach. We used linear or logistic regression models to test the interaction between study arms and these factors. We highlighted the subgroups that presented differential effects regardless of the significance of p values for the intervention and subgroup interaction. These results are exploratory in nature as the study was not powered for any of the subgroup analyses, and there was no attempt to control for potential biases.

Role of Funding Source: This work was funded by a COVID supplement to the Patient‐Centered Outcomes Research Institute (PCORI) contract (CDR‐2017C3‐9720). The funding agreement ensured the authors' independence in designing the study, interpreting the data, writing, and publishing the report.

## 3 RESULTS

**FIGURE 1:**
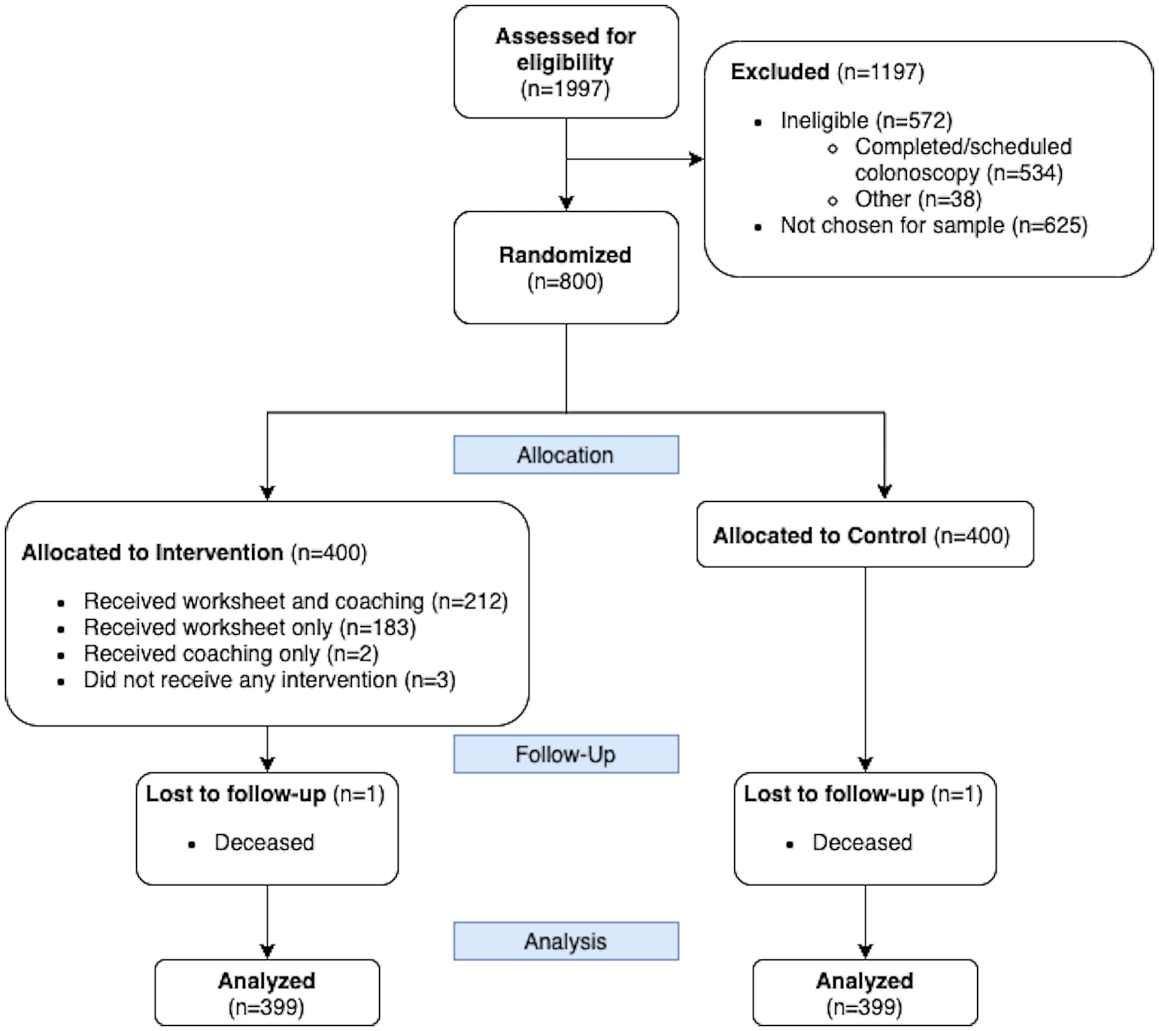
CONSORT for the screening uptake sample. The two participants who were removed had passed away before the intervention mailing for their wave.

**FIGURE 2:**
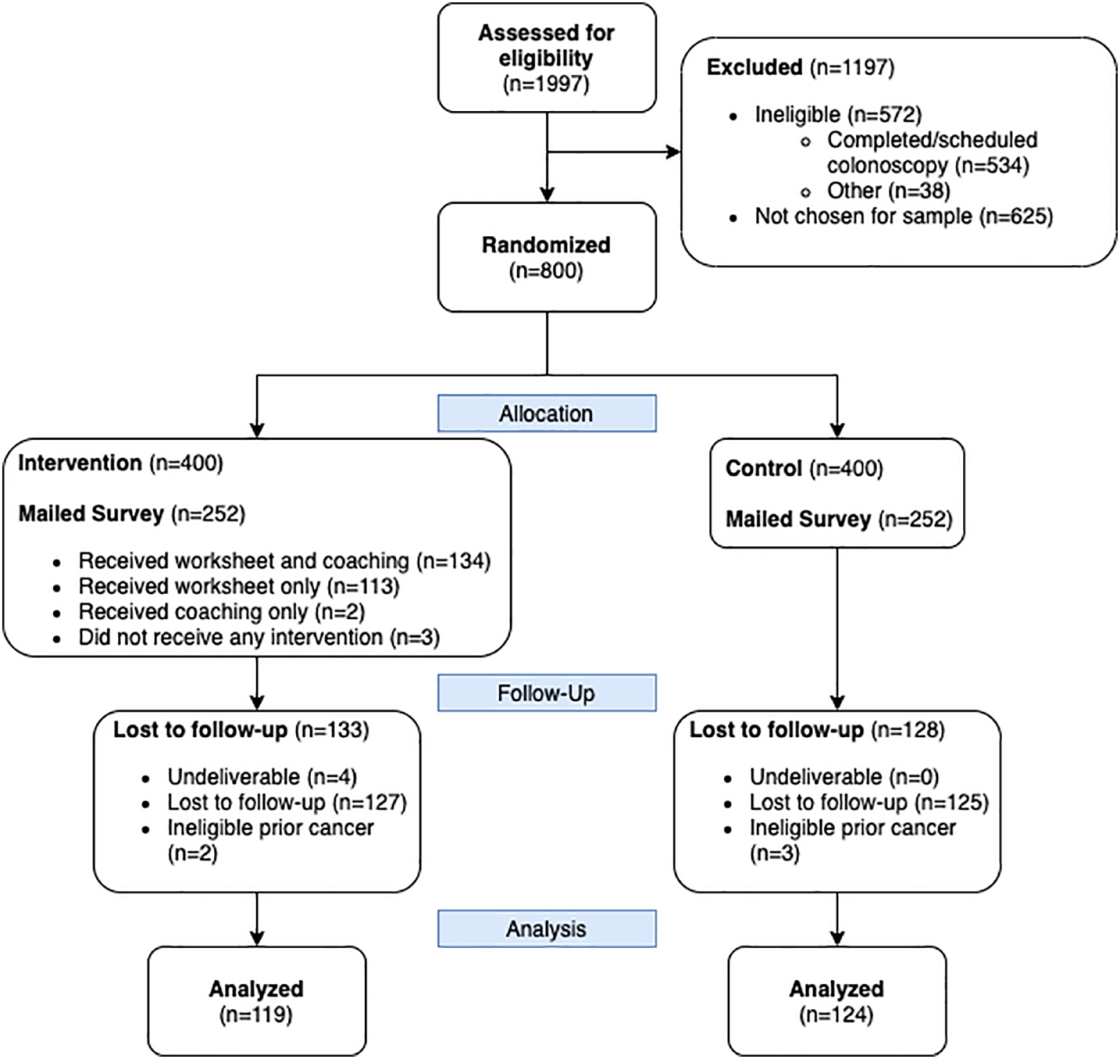
CONSORT for the sample selected for the survey

A total of 798 subjects were analyzed for the screening outcome (see Figure 1). Subjects were on average 60 years old, 53% female, and 74% non‐Hispanic White (see Table 2) and no significant differences were seen between arms. The survey response rate was 50% (248/500). The analytic sample for the survey (*n* = 243) was 61 years old, 54% female, and 70% non‐Hispanic White (see Figure 2 and Table 2). Responders to the survey tended to be slightly older than nonresponders and were similar on other variables (see Appendix 1). For the survey subsample, no significant differences were seen between arms on any other measured variables.

**TABLE 2:** Patient characteristics for the screening rate and survey analytic samples

Variable	Screening rate analytic sample		Survey analytic sample	
	Intervention arm, *N* = 399	Control arm, *N* = 399	Intervention arm, *N* = 119	Control arm, *N* = 124
Age, mean (SD)	61 (8)	60 (8)	62 (8)	60 (8)
Female, *n* (%)	217 (54)	207 (52)	69 (58)	62 (50)
Race/ethnicity^a^, *n* (%)				
White, non‐Hispanic	298 (75)	292 (73)	85 (71)	86 (69)
Hispanic	47 (12)	47 (12)	14 (12)	15 (12)
Black	18 (5)	25 (6)	1 (1)	4 (3)
Asian	14 (4)	19 (5)	2 (2)	8 (6)
Multiple or other	9 (2)	8 (2)	15 (13)	7 (6)
Unknown	13 (3)	8 (2)	2 (2)	4 (4)
Preferred language Spanish	43 (11)	42 (11)	19 (16)	20 (16)
Had coaching session	214 (54)	0 (0)	68 (57)	0 (0)
Data below collected via survey, not available on full population				
Literacy: High literacy, *n* (%)			106 (89)	105 (85)
Education^b^				
High school graduate or less			12 (10)	16 (13)
Some college or 2‐year degree			31 (27)	32 (26)
4‐year college graduate			21 (18)	28 (23)
More than a 4‐year college degree			52 (45)	45 (37)
Global health^b^, mean (SD)			51 (9)	52 (8)
Prior colonoscopies^b^				
0			14 (12)	27 (22)
1			39 (33)	35 (29)
2			30 (25)	27 (22)
3 or more			36 (30)	32 (26)
Family history of colorectal cancer			27 (23)	22 (18)
Prior polyps removed			64 (55)	69 (57)

Abbreviation: SD, standard deviation.

^a^ Race and ethnicity were taken from medical record for full sample and from patient self‐report for survey sample. Considerably more indicated multiple or other categories on self‐report than was captured in the medical record.

^b^ Missing data for education *n* = 6, global health *n* = 2, and prior colonoscopies *n* = 3.

### 3.1 Intervention exposure and interaction with healthcare team

The decision coaches reached about half of the intervention arm subjects (214/399, 54%, see Figure 1). Of the 214 reached, only 11 were referred to a GI specialist for consultation due to the complexity of patient concerns. Among the survey respondents in the intervention arm, 68% (80/117) recalled getting the decision worksheet and 38% (45/117) reported reviewing all of it. More intervention arm participants reported that they had talked with someone about CRC screening than control arm participants (56% vs. 31%, *p* < 0.001).

### 3.2 Screening rates

**FIGURE 3:**
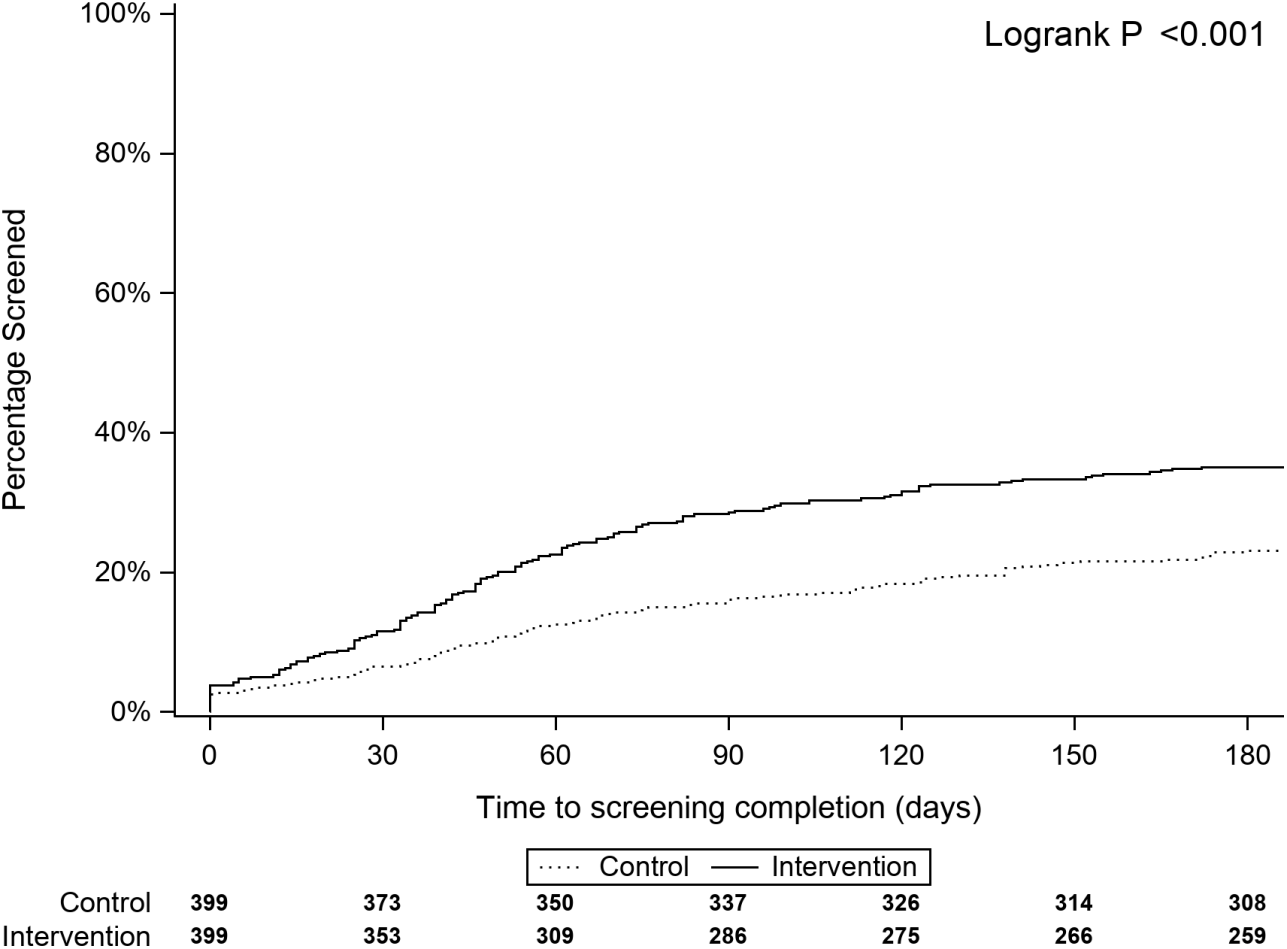
Cumulative incidence curves showing screening rates for intervention and control arm participants.

At 6 months, the intervention arm had higher screening rates than the control arm (35% vs. 23%, *p* < 0.001). The intervention arm had higher rates of colonoscopy (28% intervention vs 19% control) and stool‐based tests (7% intervention vs. 4% control). Figure 3 shows that the intervention arm completed screening earlier than patients in the control arm (median 46 days vs. 55 days, *p* < 0.001).

### 3.3 Decision‐making outcomes (survey sample only)

The intervention arm had significantly higher SDM Process scores (mean difference 0.67, 95%CI [0.41, 0.94]; *p* < 0.001) and was significantly more likely to have SURE top scores than the control arm (mean difference 21%, 95%CI [7%, 35%]; *p* = 0.003). The percentage of patients who preferred screening and who intended to follow through with screening did not vary between arms (Table 3). Of note, many respondents received a score of zero for SDM Process because they either did not speak with anyone about CRC (*n* = 136) or the interaction lacked any aspects of SDM (*n* = 23). Restricting the analyses to subjects who indicated they did talk with someone, the mean SDM Process score difference was 0.88 95%CI [0.40, 1.35], *p* < 0.001, favoring the intervention arm.

**TABLE 3:** Survey outcomes by study arms

	Control, *N* = 124	Intervention, *N* = 119	Difference [95% CI]	*p*
Shared decision‐making process score^a^ (0–4), mean (SD)	0.27 (0.67)	0.95 (1.27)	0.67 [0.41, 0.94]	<0.001
Clear preference for screening^a^, *n* (%)	78 (65)	76 (68)	3 [−10, 16]	0.75
Likely to follow through with screening, *n* (%)	56 (73)	63 (84)	11 [−3, 26]	0.14
No decisional conflict (SURE score^a^), *n* (%)	52 (48)	73 (69)	21 [7, 35]	0.003

^a^ Missing data: Shared decision‐making process score *n* = 5; clear preference *n* = 11; SURE score *n* = 27.

### 3.4 Heterogeneity analyses

Patients in the intervention arm who met with a coach had significantly higher screening rates (43% coached vs. 25% worksheet only, *p* < 0.001) and higher SDM Process scores (1.36 (SD 1.37) coached vs. 0.46 [SD 0.95], *p* < 0.001 worksheet only) than those in the intervention arm who did not connect with a coach. The intervention had a bigger impact on screening rates in patients who were not White, those with prior colonoscopies, those with better overall health, and those with more COVID worry (see Appendix 2). For SDM Process scores, the intervention was significantly more effective in females but did not appear to differ for any other variables (see Appendix 3).

## 4 DISCUSSION

To help address the massive disruption in cancer screening due to the pandemic, the investigators quickly designed and studied the impact of an SDM intervention to improve CRC screening decisions. The intervention arm achieved significantly higher screening rates at 6 months. Furthermore, the intervention survey respondents reported more SDM and had less decisional conflict than the control arm. The decision coaches were able to handle the vast majority of cases, with only 5% requiring a consult with a specialist. The study provides important new evidence of the positive impact of SDM for patients hesitant to come back for CRC screening.

The SDM intervention, a mailed decision aid worksheet followed by a coaching call, resulted in higher screening rates than usual care. A 2016 systematic review of 21 randomized trials of CRC decision aids found that the tools result in higher knowledge, greater interest in screening, and an 8% absolute increase in screening rates.^11^ The study adds to the literature demonstrating the positive impact of SDM (13% absolute increase in screening) in an overdue population where the majority had a prior colonoscopy. Importantly, the intervention was more effective in increasing screening rates for non‐White patients and those with higher COVID‐19 worry. As COVID‐19 disproportionately impacted racial and ethnic minorities,^23^ deploying interventions that support more vulnerable populations is crucial to prevent widening disparities in CRC.

Although the intervention had significantly higher SDM scores than the control arm, the absolute scores for both arms were low. In part, the low scores were due to the large number of respondents who reported no discussion of CRC screening (thus receiving a score of “0”). However, the literature typically finds cancer screening discussions (whether breast, colon, or prostate) fall short in many SDM elements.^9^, ^24^, ^25^ More complex decisions, such as surgical decisions, often have much higher SDM Process scores (>2 points out of the 4‐point scale).^18^ A higher intensity intervention is likely needed to achieve higher SDM scores in a cancer screening setting.

Planned subgroup analyses for this study found the coaching call was particularly effective in increasing screening uptake (20% absolute increase over controls). Patient navigators have been shown to increase screening rates by about 18%,^26^, ^27^ a magnitude similar to that found in this study. However, patient navigators often focus on overcoming barriers to completing scheduled tests and do not engage in the presentation of options or elicitation of patients' preferences. Given the strong, positive impact of decision coaching in this setting, a key area for future research would be examining the potential synergy for incorporating SDM more directly into patient navigation.

While the majority of patients in the study who were screened had a colonoscopy, about 20% used a stool‐based test. Stool‐based tests are a reasonable and effective option for routine screening, and during the pandemic, these tests were used in patients at higher risk of CRC due to the challenges of accessing colonoscopy.^28^, ^29^ Programs that use mass mailing of stool tests have consistently demonstrated an increase in CRC screening uptake of around 20%.^26^, ^30^ To be effective, these programs require sending multiple stool tests and following up with several phone reminders. The relative cost‐effectiveness of the direct mailing of stool tests compared to an SDM strategy is not known, and whether a combination of these approaches would be superior to either alone, is another potential area for future research.

There are several limitations to this study. First, generalizability may be limited due to the setting (single site in Boston that served as major COVID‐19 treatment center) and the patient sample (highly educated and with higher than average CRC risk). It is notable that planned subgroup analyses found the intervention to be equally effective regardless of education level and *more* effective in non‐White population. Second, the survey response rate was lower than expected. Although we found little to no difference between arms or between responders and nonresponders, it is possible the survey responses are not generalizable across the full sample. Third, coaches only made one call attempt, which improved scalability, but limited the reach and impact of the intervention which possibly resulted in an underestimate of the intervention's potential impact. Fourth, we did not conduct a formal cost‐effectiveness analysis and future work would benefit from evaluating the potential benefits of the intervention (e.g., cancers prevented and CRC mortality reduction) in light of the potential costs (e.g., complications of colonoscopy and cost of delivering intervention). Finally, the study focused on cancelations due to COVID‐19, which hopefully will not be a common occurrence. While the motivation for this study was prompted by COVID‐19, it is likely that an SDM approach that targets patients who are hesitant to complete CRC screening will be applicable even after the pandemic has subsided.

Shared decision‐making focuses on tailoring care to patients' informed preferences. In this sample, a brief SDM intervention increased the conversations about CRC screening, reduced decisional conflict, and resulted in higher screening rates. Proactive efforts are needed to re‐engage patients who had a colonoscopy canceled or delayed due to COVID‐19 to complete recommended preventive healthcare behaviors. This study suggests health systems may increase adherence to routine screening and surveillance colonoscopies with a simple mailed decision worksheet and phone follow‐up by nonclinical staff trained in decision coaching.

## FUNDING INFORMATION

This work was funded by a COVID supplement to the Patient‐Centered Outcomes Research Institute (PCORI) contract (CDR‐2017C3‐9720). The funding agreement ensured the authors’ independence in designing the study, interpreting the data, writing, and publishing the report.

## CONFLICT OF INTEREST

All authors report grant support from PCORI (CDR‐2017C3‐9270) for the study activities. Dr. Atlas reports a grant from National Cancer Institute and Dr. Richter reports approximately 25% of income derived from performing colonoscopy. No other authors have any other relationships or conflicts to report.

## ETHICAL APPROVAL STATEMENT

All study activities were reviewed and approved by the Mass General Brigham Institutional Review Board (activation date 6/2/2020).

## INFORMED CONSENT

All study activities were reviewed and approved by the Mass General Brigham Institutional Review Board. For this minimal risk study, consent was implied by the return of a completed survey. The study was conducted in accordance with the Declaration of Helsinki and the International Conference on Harmonization Guidelines for Good Clinical Practice.

## Data Availability

To promote research replicability, transparency, and future use of the data, de‐identified data sets will be created and will be available to outside researchers. Before study results have been published, researchers may request access to the data from the corresponding author and will need to provide evidence of human subjects approval and complete a data use agreement. After the study results have been published, a de‐identified data set and codebook will be deposited in an open access service such as ICPSR (https://www.icpsr.umich.edu/icpsrweb/).

## APPENDIX 1 Survey responder and nonresponder analysis

**Table:**

	b	OR 95% CI	*p*
(Intercept)	−1.35	0.26 [0.06, 1.05]	0.06
Sex‐Male	0.00	1.00 [0.70, 1.43]	0.99
Age	0.02	1.02 [1.00, 1.05]	0.04
Language‐Spanish	−0.25	0.78 [0.41, 1.45]	0.43
Study Arm‐Intervention	−0.07	0.93 [0.66, 1.33]	0.71
Wave 2	0.02	1.02 [0.62, 1.70]	0.93
Wave 3	−0.26	0.77 [0.46, 1.30]	0.34
Wave 4	0.01	1.01 [0.56, 1.83]	0.96

Abbreviations: CI, confidence interval; OR, odds ratio.

## APPENDIX 2 Results of heterogeneity analyses for screening completed at 6‐month follow‐up

**Table:**

	*n*	Usual care	Intervention	Difference (95% CI)	*p*	Interaction *p* ^a^
Age						
<61	410	24 (50/212)	34 (68/198)	0.11 (0.02, 0.20)	0.02	0.69
≥61	388	24 (42/187)	40 (72/201)	0.13 (0.04, 0.23)	0.006	
Gender						
Female	424	21 (44/207)	36 (79/217)	0.15 (0.06, 0.24)	<0.001	0.29
Male	374	27 (48/192)	37 (61/182)	0.09 (−0.01, 0.18)	0.09	
Race/ethnicity						
White, non‐Hispanic	590	26 (76/292)	36 (107/298)	0.10 (0.02, 0.18)	0.009	0.16
All others	208	15 (16/107)	33 (33/101)	0.18 (0.05, 0.30)	0.004	
Variables below limited to survey sample						
Prior colonoscopy						
None	41	15 (4/27)	7 (1/14)	−0.08 (−0.32, 0.17)	0.84	0.26
1 or more	199	38 (36/94)	51 (54/105)	0.13 (−0.02, 0.28)	0.09	
Education						
Less than college	58	20 (6/30)	32 (9/28)	0.12 (−0.14, 0.38)	0.45	0.86
College or more	179	37 (34/91)	50 (44/88)	0.13 (−0.03, 0.28)	0.12	
Health literacy						
Low	32	21 (4/19)	38 (5/13)	0.17 (−0.21, 0.56)	0.50	0.67
Moderate to high	211	35 (37/105)	47 (50/106)	0.12 (−0.02, 0.26)	0.11	
Global health score						
Low	128	30 (19/64)	38 (24/64)	0.08 (−0.1, 0.26)	0.45	0.32
High	113	36 (21/59)	57 (31/54)	0.22 (0.02, 0.42)	0.02	
COVID worry						
Low	92	44 (19/43)	43 (21/49)	−0.01 (−0.23, 0.20)	1.00	0.15
Moderate to high	136	27 (20/73)	44 (28/63)	0.17 (0.00, 0.36)	0.06	

^a^ *p* for the interaction between categories.

## APPENDIX 3 Results of heterogeneity analyses for shared decision‐making process scores

**Table:**

	*n*	Usual care, M (SD)	Intervention, M (SD)	Difference [95% CI]	*p*	Interaction *p*
Age						
<61	117	0.26 (0.66)	0.92 (1.25)	0.66 [0.28, 1.04]	0.001	0.95
≥ 61	121	0.29 (0.69)	0.97 (1.3)	0.68 [0.31, 1.05]	<0.001	
Sex						
Female^a^	128	0.27 (0.62)	1.2 (1.39)	0.93 [0.56, 1.3]	<0.001	0.02
Male	110	0.28 (0.72)	0.6 (1.01)	0.32 [0, 0.65]	0.05	
Race and ethnicity						0.45
White, non‐Hispanic	174	0.26 (0.62)	0.89 (1.16)	0.62 [0.35, 0.9]	<0.001	
All others	64	0.30 (0.78)	1.15 (1.6)	0.85 [0.17, 1.53]	0.01	
Prior colonoscopy						
None	41	0.44 (0.96)	1.14 (1.56)	0.7 [−0.1, 1.5]	0.08	0.98
1 or more	194	0.23 (0.57)	0.92 (1.23)	0.69 [0.42, 0.96]	<0.001	
Education						
Less than college^a^	56	0.28 (0.78)	0.96 (1.26)	0.68 [0.12, 1.23]	0.02	0.81
College or more	177	0.28 (0.64)	0.89 (1.23)	0.61 [0.31, 0.9]	<0.001	
Health literacy						
Low^a^	32	0.34 (0.85)	0.77 (1.15)	0.43 [−0.29, 1.15]	0.24	0.47
Not low	206	0.26 (0.64)	0.97 (1.29)	0.71 [0.43, 0.99]	<0.001	
Global health score						
Low	126	0.23 (0.66)	0.91 (1.22)	0.68 [0.34, 1.03]	<0.001	1.00
High	110	0.33 (0.69)	1.01 (1.34)	0.68 [0.27, 1.1]	0.002	
COVID worry						
Somewhat/not at all	92	0.3 (0.73)	1.05 (1.36)	0.75 [0.3, 1.2]	0.001	0.55
Extremely/moderately	132	0.28 (0.67)	0.87 (1.17)	0.58 [0.24, 0.92]	0.001	

^a^ These analyses met the assumption for equal variances and were analyzed with t‐tests. All other analyses used a Welch two‐sample *t*‐test.
